# Suppressive Potential of *Paenibacillus* Strains Isolated from the Tomato Phyllosphere against Fusarium Crown and Root Rot of Tomato

**DOI:** 10.1264/jsme2.ME13172

**Published:** 2014-06-10

**Authors:** Ikuo Sato, Shigenobu Yoshida, Yutaka Iwamoto, Masataka Aino, Mitsuro Hyakumachi, Masafumi Shimizu, Hideki Takahashi, Sugihiro Ando, Seiya Tsushima

**Affiliations:** 1 Environmental Biofunction Division, National Institute for Agro-Environmental Sciences, 3–1–3 Kan-nondai, Tsukuba, Ibaraki 305–8604, Japan; 2 Hyogo Prefectural Technology Center for Agriculture, Forestry and Fisheries, Kou 1533 Minaminooka, Beppucyo, Kasai, Hyogo 679–0198, Japan; 3 Faculty of Applied Biosciences, Gifu University, 1–1 Yanagido, Gifu, Gifu 501–1193, Japan; 4 Graduate School of Agricultural Science, Tohoku University, 1–1 Amamiya-machi, Tsutsumidori, Aoba-ku Sendai, Miyagi 981–8555, Japan; 5 Natural Resources Inventory Center, National Institute for Agro-Environmental Sciences, 3–1–3 Kan-nondai, Tsukuba, Ibaraki 305–8604, Japan

**Keywords:** Fusarium crown and root rot, *Fusarium oxysporum* f. sp. *radicis-lycopersici*, Phyllosphere bacteria, *Paenibacillus*, induced resistance

## Abstract

The suppressive potentials of *Bacillus* and *Paenibacillus* strains isolated from the tomato phyllosphere were investigated to obtain new biocontrol candidates against Fusarium crown and root rot of tomato. The suppressive activities of 20 bacterial strains belonging to these genera were examined using seedlings and potted tomato plants, and two *Paenibacillus* strains (12HD2 and 42NP7) were selected as biocontrol candidates against the disease. These two strains suppressed the disease in the field experiment. Scanning electron microscopy revealed that the treated bacterial cells colonized the root surface, and when the roots of the seedlings were treated with strain 42NP7 cells, the cell population was maintained on the roots for at least for 4 weeks. Although the bacterial strains had no direct antifungal activity against the causal pathogen *in vitro*, an increase was observed in the antifungal activities of acetone extracts from tomato roots treated with the cells of both bacterial strains. Furthermore, RT-PCR analysis verified that the expression of defense-related genes was induced in both the roots and leaves of seedlings treated with the bacterial cells. Thus, the root-colonized cells of the two *Paenibacillus* strains were considered to induce resistance in tomato plants, which resulted in the suppression of the disease.

Fusarium crown and root rot (FCRR) of tomato is caused by *Fusarium oxysporum* f. sp. *radicis-lycopersici* (FORL), which is a disease that is commonly observed in locations worldwide, such as Europe, North America, and Japan (15, 38, 47). The symptoms of the disease consist of the browning and rotting of the crown and roots and the yellowing of leaves. Advanced symptoms are wilting and death (32), which leads to a loss in fruit yield. The fruit yield loss due to the disease in Florida, USA was reported to range between 15% and 65% (32). Although attempts to control this challenging disease in Japan have included physical and chemical approaches, such as soil solarization and the use of chemical fumigants, respectively, no biological approaches such as the use of microbes antagonistic to the causal fungi are currently available. The development of biological controls has recently received attention because of their economic and ecological advantages, as well as political demands. Several studies have examined biological control approaches against the disease (6, 28, 31, 35). In Japan, Iwamoto and Aino (20) reported that a commercial biocontrol agent (an endophytic bacterium of tomato roots, *Pseudomonas fluorescens* FPH9601) significantly suppressed the disease in tomato fields, although the sale of the agent is currently suspended. Another study on disease suppression indicated the successful combined use of a plant growth-promoting fungus (PGPF), *Fusarium equiseti*, and biodegradable pots in fields (16); however, commercial products have not yet been registered. Therefore, more novel microbial agents that are applicable to the actual practices used in tomato cultivation need to be developed.

To develop biocontrol agents based on microbial colonization and propagation principles, selection from the resident microbes of the target plant is preferable (44). Thus, microbes colonizing tomato plants are good candidates for biocontrol agents of the disease. Based on this principle, several studies on the control of the disease have been demonstrated using various microorganisms (2, 3, 6). Kamilova *et al.* (22) reported that a *Pseudomonas fluorescens* strain and *Pantoea agglomerans* strain selected using an enrichment method were superior root colonizers that could significantly control the tomato disease caused by FORL, which demonstrated the importance of the colonization ability of microbes for biocontrol. Although selection from resident microbes inhabiting roots appears to be more feasible for tackling the *Fusarium* disease in the tomato, we hypothesized that novel and unique microbial candidates may be obtained as biocontrol agents from the aboveground parts of the plants (*i.e.*, the phyllosphere). Root-inhabiting microbes have a higher ability to colonize plants *per se* (46) and several of these microbes have the potential to suppress plant diseases (44, 45), whereas few attempts have been made to use phyllosphere microbes against soilborne diseases. In addition, soil is recognized to be a significant source of phyllosphere microbes (46), which suggests their potential to have suppressive functions even in soils or the rhizosphere.

The first objective of this study was to evaluate the suppressive potential of phyllosphere microbes in the tomato against FCRR, which could then be considered useful as biocontrol agents. We previously isolated and preserved culturable microbes inhabiting various plant surfaces (8, 9, 37, 49, 51). On tomato plants, Enya *et al.* (8, 9) reported the taxonomical grouping of 2138 culturable bacterial strains isolated from a greenhouse- and field-grown tomato phyllosphere as well as their antifungal activities against several aboveground tomato diseases. Of the microbial stocks isolated from the tomato phyllosphere, emphasis was placed on *Bacillus* and *Paenibacillus* in this study because members of these bacterial groups contribute to crop production (27) and produce endospores that are tolerant to heat and desiccation (44), leading to the easy and costless generation of the final commercial products. Several commercial products originating from these bacterial groups have already been developed (11). Once two *Paenibacillus* strains from the tomato phyllosphere were verified to have suppressive potentials, we determined the mode of action for the suppressive abilities of the *Paenibacillus* strains.

## Materials and Methods

### Tomato plants

The tomato plant (*Solanum lycopersicum* Mill.) cv. House-momotaro (Takii Seed, Kyoto, Japan) was used in this experiment. The seeds were sown in each cell (3×3×4.5 cm) of a plastic 128-cell tray (Takii Seed) containing commercial soil (0.1 g N, 1.25 g P, 0.1 g K, and 0.1 g Mg kg^−1^; Ryousaibaido Pp; Nihon Hiryo, Tokyo, Japan), grown in a greenhouse at 23°C for 3 weeks, and then used for seedling or potted plant experiments as described below.

### Bacterial strains

The 13 *Bacillus* and 7 *Paenibacillus* strains summarized in Table 1 were used in this study. They were isolated from the healthy leaves of field or greenhouse-grown tomatoes in Tsukuba, Japan in 2003 (8) and preserved in the National Institute for Agro-Environmental Sciences, Tsukuba. These strains were chosen as representatives belonging to each cluster in the phylogenetic tree comprising 190 *Bacillus* and *Paenibacillus* strains, as discussed in a previous study (9).

### Preparation of the *F. oxysporum* f. sp. *radicis-lycopersici* (FORL) inoculum

FORL strain For6-3 was isolated from greenhouse-grown tomato roots (20) and preserved in the Hyogo Prefectural Technology Center for Agriculture, Forestry and Fisheries (Kasai, Japan) for use in the present study. For6-3 was shaken (120 rpm) in potato dextrose liquid medium (Difco Laboratories Inc., Detroit, MI, USA) for 5 d at 28°C in the dark, and the culture was filtered through cheesecloth to remove mycelial fragments. The resulting budcells were then harvested by centrifugation at 4,000×*g* for 10 min, washed twice with sterilized distilled water (SDW), suspended in SDW to achieve a concentration of 1.0×10^7^ budcells ml^−1^, and then used in the seedling experiment. In pot experiments, 40 mL of the prepared budcell suspension was poured into 600 g of an autoclaved soil– wheat bran medium (a mixture of clay soil and wheat bran at a ratio of 4:1) in a plastic case (299×224×62 mm) and incubated for 2 weeks at 25°C in the dark. After incubation, fungal propagules grown in the medium were ground using a mortar and stored at 4°C until use. The fungal density in the medium was checked by a dilution plating method before use and confirmed to range between 3 and 8×10^7^ CFU g^−1^ of medium.

### Seedling experiment

To screen bacterial strains having a suppressive effect on FCRR, each *Bacillus* and *Paenibacillus* strain was shaken (120 rpm) in R2A liquid medium (Wako Pure Chemical Industries, Osaka, Japan) for 48 h at 28°C in the dark, collected by centrifugation at 6000×*g* for 10 min, washed twice, and suspended in SDW to achieve an OD_600_ =0.5. Five milliliters of the bacterial suspension of each strain was drenched into the soil of each seedling with a pipette, and 5 mL of SDW was similarly applied to another eight seedlings as a control. Two d after the bacterial treatment, 15 mL of the budcell suspension of FORL For6-3 prepared was inoculated into the soil of each seedling with a pipette. After incubation for 4 weeks in a greenhouse at 23°C, the soil was carefully dug away from the roots of the seedlings and the roots were thoroughly washed with tap water. The degree of root rot was then evaluated based on a lesion index value of 0, 1, 2, 3, 4, or 5 (0, no lesion; 1, lesion areas on 1/10 to 1/4 of the root area; 2, lesion areas on >1/4 to 1/3 of the root area; 3, lesion areas on >1/3 to 2/3 of the root area; 4, lesion areas on >2/3 of the root area; 5, dying). The disease severity (DS) and protective value (PV) were calculated based on the following formulas: DS = (0n_0_ + 1n_1_ + 2n_2_ + 3n_3_ + 4n_4_ + 5n_5_)/5(n_0_ + n_1_ + n_2_ + n_3_ + n_4_ + n_5_) × 100, where n_0–5_ is the number of seedlings with each of the 1–5 lesion index values; PV = 100 − ([DS of the sample/DS of the control] × 100). The calculation was performed using eight replicated seedlings and the experiment was repeated four times. Results were analyzed by ANOVA followed by Fisher’s LSD test using Kaleida-Graph (Synergy Software, PA, USA)

### Potted plant experiment

Bacterial suspensions of the strains 12HD2 and 42NP7, which displayed suppressive effects in the seedling experiment, were again applied to each of the eight seedlings using the same procedure, except for bacterial suspensions achieving an OD_600_=0.3. SDW-treated seedlings were also prepared as a control. Two d after the bacterial treatment, the seedlings were individually transplanted to vinyl pots 9 cm in diameter that contained FORL-contaminated soil, which was prepared by mixing sieved and air-dried clay soil and a commercial soil (Engeibaido-1-gou; Nihon Hiryo, Tokyo, Japan) at a ratio of 3:1, followed by mixing with an appropriate amount of FORL propagules in a soil–wheat bran medium, to reach a final concentration of 1.0×10^5^ CFU g^−1^ soil. After transplanting, the pots were placed in a greenhouse for 4 weeks at 23°C, and the degree of root rot on each potted tomato plant was then evaluated. The DS was calculated using the same procedures as those in the seedling experiment. The calculation was performed using eight replicated seedlings and the experiment was repeated three times. Results were subjected to ANOVA followed by Fisher’s LSD test as described above. To estimate the FORL density in the root tissues of the potted plants, 1 g (fresh weight) of the roots of a representative potted plant, with an average severity of root rot among the eight plants treated with each bacterial strain, was excised from the middle of the roots using sterilized scissors. The roots were surface-sterilized in 70% ethanol for 1 min and then rinsed twice with 10 mM potassium phosphate buffer (pH 7.4). Roots obtained from uninoculated healthy plants were prepared in the same way to act as negative controls. The roots were subsequently ground in a mortar with 10 mL of the potassium phosphate buffer, diluted appropriately, and spread on agar plates of Komada’s selective medium (23). This medium specifically detects nonpathogenic and pathogenic *F. oxysporum*, including FORL and other formae specialists. After 10 d of incubation at 25°C, the number of presumable FORL colonies on each plate was counted and the FORL density (CFU g^−1^ roots) was calculated based on these counts. The FORL recovery experiment was repeated three times. Results were analyzed by ANOVA followed by Fisher’s LSD test as described above.

### Field experiment

A field experiment on biological control for FCRR was carried out in a plastic greenhouse at the experimental farm of Hyogo Prefectural Technology Center for Agriculture, Forestry and Fisheries, Japan in 2013. A FORL-contaminated field was prepared by mixing the soil and FORL propagules grown on barley grain medium at a ratio of 24 kg dry weight are^−1^ (20), and a chemical fertilizer S604 (160 g N, 100 g P, 140 g K kg^−1^; JCAM AGRI, Tokyo, Japan) was also applied to the field soil at the ratio of 4 kg are^−1^. The tomato seedlings were individually grown in vinyl pots 9 cm in diameter that contained Sumi soil N-100 (0.1 g N, 0.25 g P, 0.1 g K l^−1^; Sumika Agrotech, Osaka, Japan) and grown for 8 weeks. Thirty milliliters of the bacterial suspension of the strains 12HD2 and 42NP7, prepared as described above, were poured into the soil of each plant. SDW-treated plants were also prepared as a control. Five d after the treatments, the plants were transplanted in mid-February 2013 in two rows with 30 cm between plants in a row and with 120 cm of a ridge width. Each plot (2 m^2^) included 9 plants and 6 replicated plots per treatment were systematically distributed in the field. After transplanting, the plants were cultivated in the field for 18 weeks, and the degree of root rot on each placed tomato plant was then evaluated. The DS in each plot was calculated using the same procedures as in the seedling experiment. Results were subjected to ANOVA followed by Fisher’s LSD test as described above.

### The suppressive effect of bacterial cells and culture filtrate of strains 12HD2 and 42NP7

Bacterial strains 12HD2 and 42NP7 were shaken in R2A, a cell suspension of each strain was prepared by the centrifugation method described above, and a culture filtrate of each strain following centrifugation was obtained. The cell suspension and culture filtrate of the strains were individually applied to the soils of eight seedlings in a cell tray. Five milliliters of SDW and R2A were also applied to the soils of another eight seedlings, as controls for the cell suspension and culture filtrate, respectively. FORL inoculation and the subsequent DS calculations were based on the degree of root rot and performed using the same procedures as those used in the seedling experiment. The experiment was repeated three times, and results were analyzed by ANOVA followed by Fisher’s LSD test.

### *In vitro* antifungal activity

The direct antifungal activities of the 12HD2 and 42NP7 strains were evaluated using two nutrient media, PDA or 1.5% agar-supplied R2A (AR2A) plates containing FORL budcells. One ml of the FORL For6-3 budcell suspension (1×10^7^ mL^−1^) was mixed with 10 mL of PDA or AR2A at 55°C, and the mixture was immediately poured into a petri dish with a diameter of 9 cm. After solidification, paper discs (8 mm diameter) immersed with each cell suspension of the 12HD2 and 42NP7 strains were placed onto the plates. Paper discs immersed with a cell suspension of *Escherichia coli* DH5α grown on Luria–Bertani (LB) medium and iturin-producing *Bacillus amyloliquefaciens* RC-2 (48) grown on R2A were also placed onto the plates as negative and positive controls, respectively. Antifungal activity was determined by the appearance of growth inhibition zones around the paper disks after incubation for 3 d (PDA plate) or 10 d (AR2A plate) at 28°C in the dark.

### Scanning electron microscopy

To obtain semi-aseptic tomato seedlings, seeds of cv. House-momotaro were surface-sterilized by immersion in 70% ethanol for 1 min and 5% antiformin for 5 min, followed by rinsing twice with SDW. After eliminating the extra water on the sterilized filter paper, the sterilized seeds were sown in a cell tray containing commercial soil (Ryousaibaido Pp) that had been autoclaved twice for 20 min at 121°C and placed in a growth chamber for 2 weeks at 23°C under fluorescent light (340 μmol m^−2^ s^−1^) with a photoperiod of 14 h (light)/10 h (dark) to grow seedlings. A cell suspension (5 mL) of 12HD2 and 42NP7 prepared as described above and 5 mL SDW (as a control) were each applied to each soil of the seedlings. After incubation in the growth chamber for 4 d, the soil was carefully dug away from the roots of the seedlings and the roots were washed by shaking thoroughly in SDW. The roots were immediately cut out, divided into small segments, and fixed in 2% (v/v) glutaraldehyde in 0.1 M phosphate buffer (pH 7.4) for 1 d at 4°C. The fixed samples were then passed through increasing concentrations of ethanol (30%, 50%, 70%, 90%, 95%, and 100% [v/v]). The dehydrated samples were subsequently dried, spattered with gold–palladium, and observed with a scanning electron microscope (SEM) (JSM- 5610LV; JEOL Tokyo, Japan) using a previously reported procedure (50).

### Enumeration of strain 42NP7 on roots of tomato seedlings

Strain 42NP7rk, a spontaneous mutant of strain 42NP7, which is resistant to both rifampicin and kanamycin, was obtained on AR2A containing both 50 μg mL^−1^ antibiotics and used for the experiment. No significant differences were observed in the growth rates or nutrient availability between the mutant strain and wild type (data not shown). Although attempts were made to generate mutants of strain 12HD2 using several similar procedures, no mutated strains were obtained. Strain 42NP7rk was cultured on AR2A plates containing 50 μg mL^−1^ each of rifampicin and kanamycin for 4 d at 28°C in the dark, then cells on the plates were harvested and suspended in SDW after washing by centrifugation at 6000×g for 10 min. After the concentration of the cell suspension was adjusted to 6.7×10^7^ CFU mL^−1^, aliquots (5 mL) of the cell suspension were inoculated into the soil of each tomato seedling grown in the cell tray, using the same procedures for the above seedling experiment as described above. Seedlings that were not inoculated were also prepared as controls. The inoculated seedlings were incubated for 0 (*i.e.* immediately after the inoculation), 3, 7, 14, 21, or 28 d in the greenhouse at 23°C, were then carefully plucked from the cell tray, and the surface of the roots were thoroughly washed in tapped distilled water using a sterilized artist brush to remove the attached small soil particles. After extra water was absorbed using a paper towel, the washed roots (fresh weight: 0.14–0.52 g) of each seedling were cut into small segments using a scissors and ground in a mortar with 10 mM phosphate buffer (pH 7.0) (5 mL per g of root segments). Appropriate dilutions of the obtained macerates were spread onto AR2A plates containing 50 μg mL^−1^ of both antibiotics. Colony counts were carried out 7 d after the incubation of plates at 28°C in the dark. Two seedlings were used for the bacterial enumeration at each recovery.

### Antifungal activities of the extracts from roots treated with the bacterial cells of strains 12HD2 and 42NP7

Potted tomato plants treated with each bacterial suspension and transplanted into the 9-cm pot containing FORL-infested soil were prepared, as described above in the potted plant experiment, to ascertain antifungal activity in the tomato roots following the treatment with the bacterial strains 12HD2 and 42NP7. Similarly, potted tomato plants treated with SDW in the infested soils (SDW-treated roots) or those without the treatment and grown in uninfested soils (untreated roots) were also prepared as controls. Four weeks after transplanting, the DS values in the treated and untreated roots were evaluated as described above, the roots were then cut out using sterilized scissors (approximately 5.03–10.12 g fresh weight), transferred into glass petri dishes, and immersed in 50 mL acetone for 30 min at room temperature. After passing through filter paper to remove debris, each acetone extract was air-dried and dissolved with 50% methanol at a concentration of 60 mg mL^−1^ (dry weight). Aliquots (10 μL) of the concentrated extracts were each placed onto a small mycelial block (approximately 1 mm^3^) of FORL For6-3 cultured on 1.5% agar plates following the procedures used in a previous study (48). Ten microliters of 50% methanol was similarly placed onto another mycelial block as a relative control. To ascertain the activity against a non-tomato pathogen, *Colletotrichum dematium* S9733, the causal fungus of mulberry anthracnose, was also examined on PDA (Difco Laboratories Inc.) plates. This fungal species was chosen because it was assumed to be more susceptible to antibiotics based on the findings of a previous study (48). After incubation for 1 d with For6-3 and 2 d with S9733 at 25°C, the diameter of the mycelial colony that developed in each mycelial block was measured and four replicate measurements were averaged. The antifungal activity of each extract was evaluated based on the relative averaged diameter of the mycelial colony treated with the relative control on each fungal strain, and the results were analyzed by ANOVA followed by Fisher’s LSD test.

### RT-PCR analysis

To isolate total RNA from tomato seedlings, the seeds of cv. House-momotaro were sown in plastic pots (7 cm in diameter) containing 110 g quartz sand, placed in the growth chamber for 18 d under the same conditions as described above, and fertilized with 2000-fold diluted Hyponex solution (Hyponex Japan, Osaka, Japan) at 1 d intervals. Ten milliliters of each cell suspension (OD_600_=0.3) of the 12HD2 and 42NP7 strains prepared according to the above procedures was then poured into the sand of each tomato seedling. Cell suspensions of *E. coli* DH5α prepared from the LB culture were also applied to other seedlings as a negative control. The tomato seedlings were thoroughly pulled out 2 d after the bacterial treatment, and total RNA was immediately isolated from a 0.1 g segment from the roots at 15–20 mm under the surface of the sands and a second leaf of the individual seedlings using an ISOGEN kit (Nippon gene, Tokyo, Japan) according to the instruction manual. First-strand cDNA was synthesized from total RNA using a PrimeScript RT-PCR Kit (Takara, Ohtsu, Japan) according to the instruction manual. First-strand cDNA (the equivalent of 20 ng reverse-transcribed RNA) was added to 20 μL of the PCR reaction mixture (Ex Taq; Takara) with 0.2 μM of each primer plus a negative control (non-reverse-transcripted RNA). The reaction was run with the following program: 23–30 cycles at 95°C for 20 s, 60°C for 30 s, and 72°C for 30 s. The expression levels of the following defense-related genes were monitored: *PR-1* (42) and *PR-5* (33) (encoding an acidic antifungal protein and an acidic thaumatin-like protein, respectively) are salicylic acid (SA)-responsive marker genes, and *PR-3* (5) and *PR-6* (12) (encoding a basic chitinase and basic proteinase-inhibitor, respectively) are jasmonic acid/ethylene (JA/ET)-responsive maker genes. The internal standard actin gene *Act* was also monitored. The gene-specific primers used in this study are listed in Table 2.

## Results

### Seedling experiments

A screening was performed for suppressive strains against FCRR. Although the reductions observed in the disease severity were slightly more with all strains than with the control, only two strains, 12HD2 and 42NP7, significantly (*P*<0.05) suppressed the disease. Their disease severity (DS) values were 27.5 and 22.5, respectively, while that of the control was 46.3 (Table 1). The protective values (PVs) of 12HD2 and 42NP7 corresponded to 40.6 and 51.4, respectively. Based on similarities in the 16S rRNA gene sequences, 12HD2 and 42NP7 were found to be the most closely related to *Paenibacillus alginolyticus* DSM5050 (98.6% homology) and *Paenibacillus favisporus* GMP01 (99.7% homology), respectively.

### Potted plant experiment

**Fig. 1:**
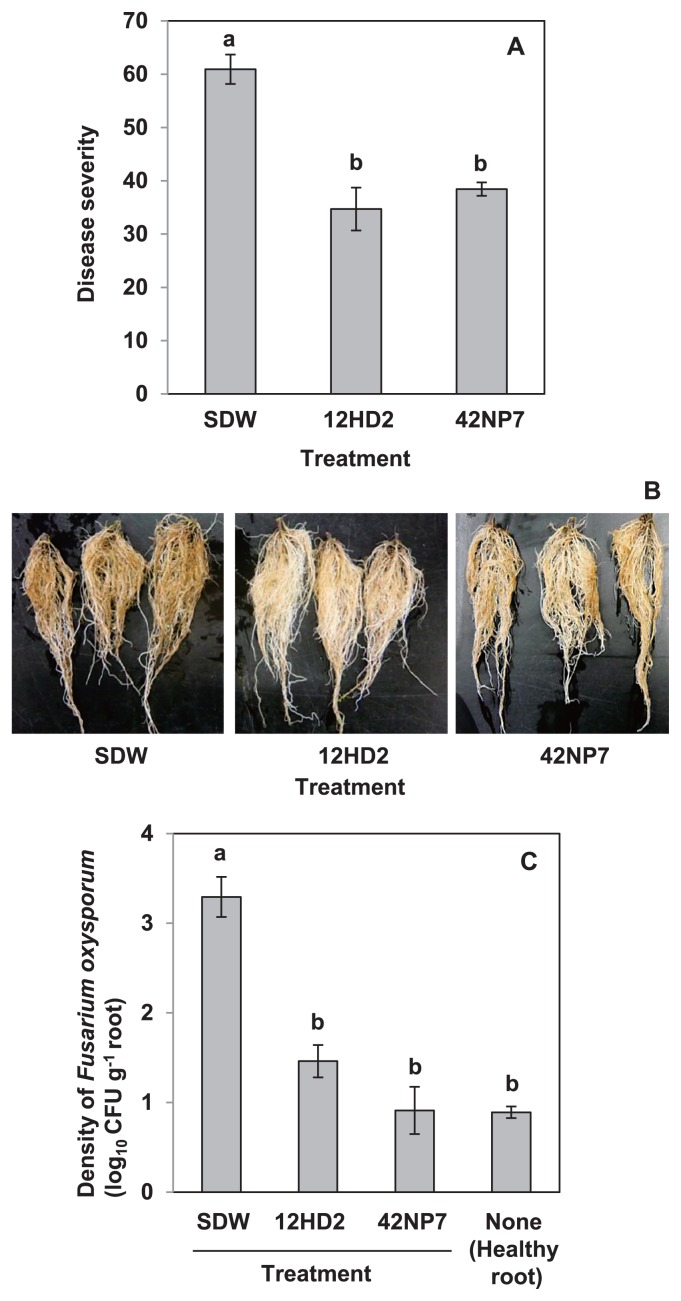
Suppressive effects against Fusarium crown and root rot by *Paenibacillus* strains 12HD2 and 42NP7 in a potted plant experiment. (A) Disease severity caused by *Fusarium oxysporum* f. sp. *radicis-lycopersici* in the roots of tomato seedlings treated with each strain. Each value indicates the mean of three experiments and the bar denotes the standard error of the mean. Different letters within each column indicate significant differences (*P*<0.05) according to Fisher’s LSD test. (B) Representatives of the washed roots of seedlings treated with SDW (left) and a cell suspension of 12HD2 (right). Note that rot symptoms were suppressed by the treatment with a cell suspension of 12HD2. (C) Density (CFU g^−1^ of root) of *Fusarium oxysporum* isolated from the roots of potted tomato seedlings treated with each strain. The fungal species were also isolated from the healthy roots of uninoculated seedlings as a negative control (Healthy roots). Each value indicates the mean of three experiments and the bar denotes the standard error of the mean. Different letters within each column indicate significant differences (*P*<0.05) according to Fisher’s LSD test.

Because the 12HD2 and 42NP7 strains were selected as positive for the suppression of FCRR in the seedling experiment, they were examined further in the potted plant experiment. Consequently, the DS values of roots treated with 12HD2 and 42NP7 were 34.7 and 38.4, respectively, and were significantly (*P*<0.05) lower than that of the sterilized distilled water (SDW)-treated control roots (DS = 60.9; Fig. 1A), with a reduction being observed in the typical browning symptoms (Fig. 1B). When the density of total *F. oxysporum* in these roots was calculated using Komada’s selective medium, the densities of roots treated with both 12HD2 and 42NP7 were significantly (*P*<0.05) lower (less than one 80th) than those of SDW-treated roots (Fig. 1C). Although the medium used could detect not only pathogenic, but also nonpathogenic strains, the densities of the fungal species isolated from uninoculated healthy roots (negative control), most of which were assumed to be nonpathogenic or species other than FORL, were approximately 10 CFU g^−1^ roots (Fig. 1C). This indicated that the majority of the colonies isolated from each bacterial strain- or SDW-treated root were FORL.

### Field experiment

**Fig. 2:**
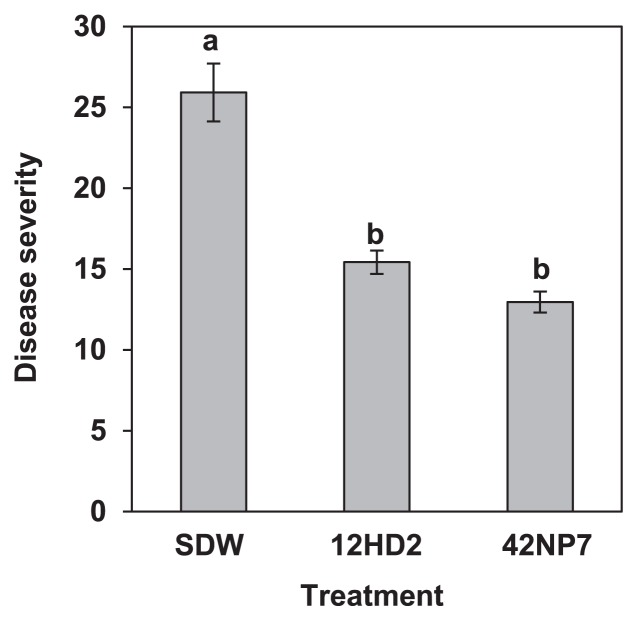
Suppressive effects against Fusarium crown and root rot by *Paenibacillus* strains 12HD2 and 42NP7 in a field experiment. Each value indicates the mean of six experiments and the bar denotes the standard error of the mean. Different letters within each column indicate significant differences (*P*<0.05) according to Fisher’s LSD test.

The suppressive strains against FCRR in the potted plant experiment, 12HD2 and 42NP7, were further examined in the field experiment. Consequently, the DS values on the roots treated with 12HD2 and 42NP7 were 15.4 and 12.9, respectively, and were significantly (*P*<0.05) lower than that of the SDW-treated control roots (DS=25.9; Fig. 2). PVs by the treatments with 12HD2 and 42NP7 were 40.4 and 50.0, respectively.

### Disease suppressive effects by the bacterial cells and culture filtrate of strains 12HD2 and 42NP7

**Fig. 3:**
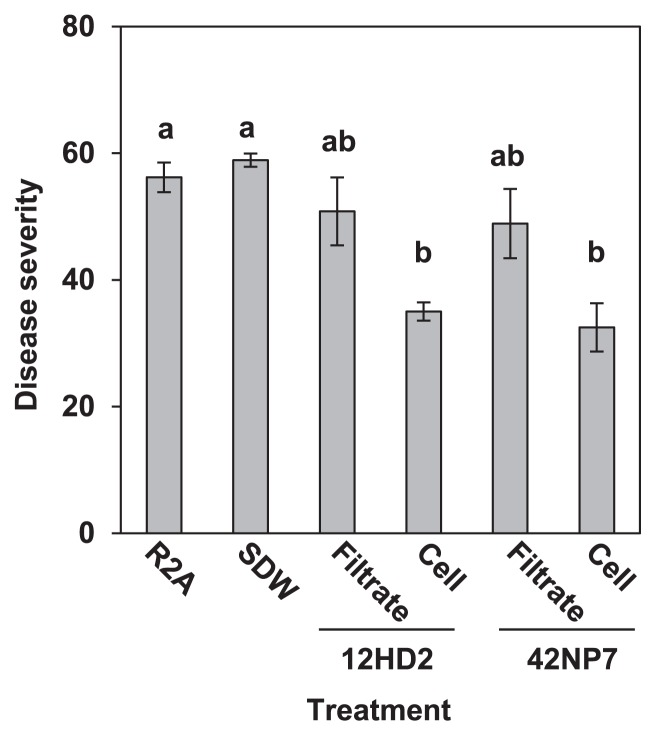
Disease severity caused by *Fusarium oxysporum* f. sp. *radicis-lycopersici* in the roots of potted tomato seedlings treated with bacterial cells and a culture filtrate of *Paenibacillus* strains 12HD2 and 42NP7. Each value indicates the mean of three experiments and bars denote the standard error of the mean. Different letters within each column indicate significant differences (*P*<0.05) according to Fisher’s LSD test.

Fig. 3 shows that the DS values on the roots treated with either cell suspension of 12HD2 or 42NP7 were significantly (*P*<0.05) lower than those with the control treatment with SDW, whereas DS values on the roots treated with the culture filtrates of either strain were not significantly different from the control value (R2A treatment).

### *In vitro* antifungal activity

**Fig. 4:**
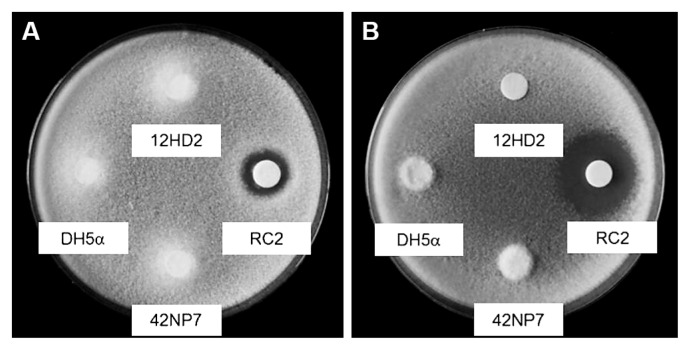
*In vitro* antifungal activities of the *Paenibacillus* strains 12HD2 and 42NP7 against *Fusarium oxysporum* f. sp. *radicis-lycopersici* on PDA (A) and 1.5% agar-supplied R2A (B) plates. Paper discs immersed with each cell suspension of strains 12HD2, 42NP7, *Escherichia coli* DH5α (negative control), and iturin-producing *Bacillus amyloliquefaciens* RC-2 (positive control) were placed on each agar plate containing budcells of *Fusarium oxysporum* f. sp. *radicis-lycopersici*. Observations were made 3 (A) and 10 (B) d after the incubation at 28°C. Note only the positive control formed clear zones, which indicated antifungal activity in both plates.

Filter disks containing a cell suspension of each *Paenibacillus* strain, 12HD2 and 42NP7, formed no clear inhibitory zones around the disks on PDA or AR2A plates containing FORL (Fig. 4). Only suspensions of *B. amyloliquefaciens* RC-2, as a positive control, formed clear zones on both plates.

### Scanning electron microscopy

**Fig. 5:**
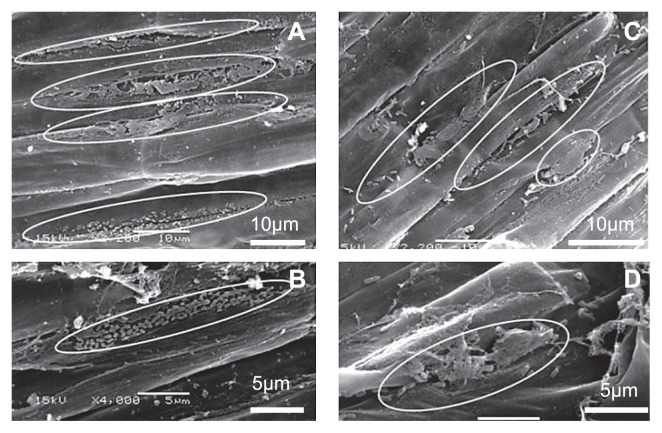
Scanning electron microscopic images of the roots of semi-aseptic tomato seedlings treated with the cells of *Paenibacillus* strains 12HD2 (A, B) and 42NP7 (C, D). The circles in A–D indicate assemblages or clusters of cells at the interspaces between epidermal cells.

Fig. 5 shows scanning electron microscopic images of tomato roots treated with a cell suspension of *Paenibacillus* strains 12HD2 (Fig. 5A, B) and 42NP7 (Fig. 5C, D). Cells of both strains colonized the surfaces, particularly at the interspaces between epidermal cells. Cells were often viewed as assemblages or clusters at the interspaces in 12HD2 cell-inoculated roots, and appeared to be embedded in an outer layer of the roots (the circles in Fig. 5A, B). 42NP7 cells also formed clusters at the interspaces (Fig. 5C). They were also observed at other locations on the root surface, presumably utilizing extracellular compounds (Fig. 5D). Similar bacterial cells and structures were not observed on the surface of the control roots.

### Enumeration of colonized strain 42NP7 on roots of tomato seedlings

**Fig. 6:**
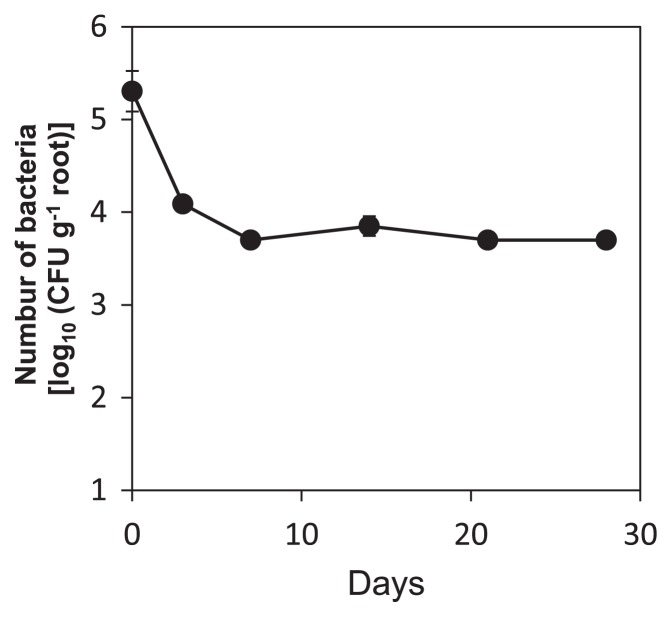
Population dynamics of 42NP7rk in the roots of the tomato after the inoculation. The cell suspension of strain 42NP7rk, the antibiotic-resistant strain of 42NP7, was inoculated into the roots of tomato seedlings and the CFU was determined. Each value indicates the mean of three experiments and bars denote the standard error of the mean.

Fig. 6 shows the dynamics of the bacterial density of strain 42NP7rk, an antibiotic-resistant mutant of strain 42NP7, inoculated to the roots of tomato seedlings. The initial density of the inoculated bacterial strain was 2.5×10^5^ CFU g^−1^ roots. Although the density in the root macerates rapidly decreased by approximately 1.2×10^4^ CFU g^−1^ roots until 3 d after the inoculation, the bacterial density afterwards was nearly flat, at least until 28 d after the inoculation. No bacterial colonies appeared from the macerates of non-inoculated control roots on the plates.

### Antifungal activities of extracts from roots treated with bacterial cells of strains 12HD2 and 42NP7

**Fig. 7:**
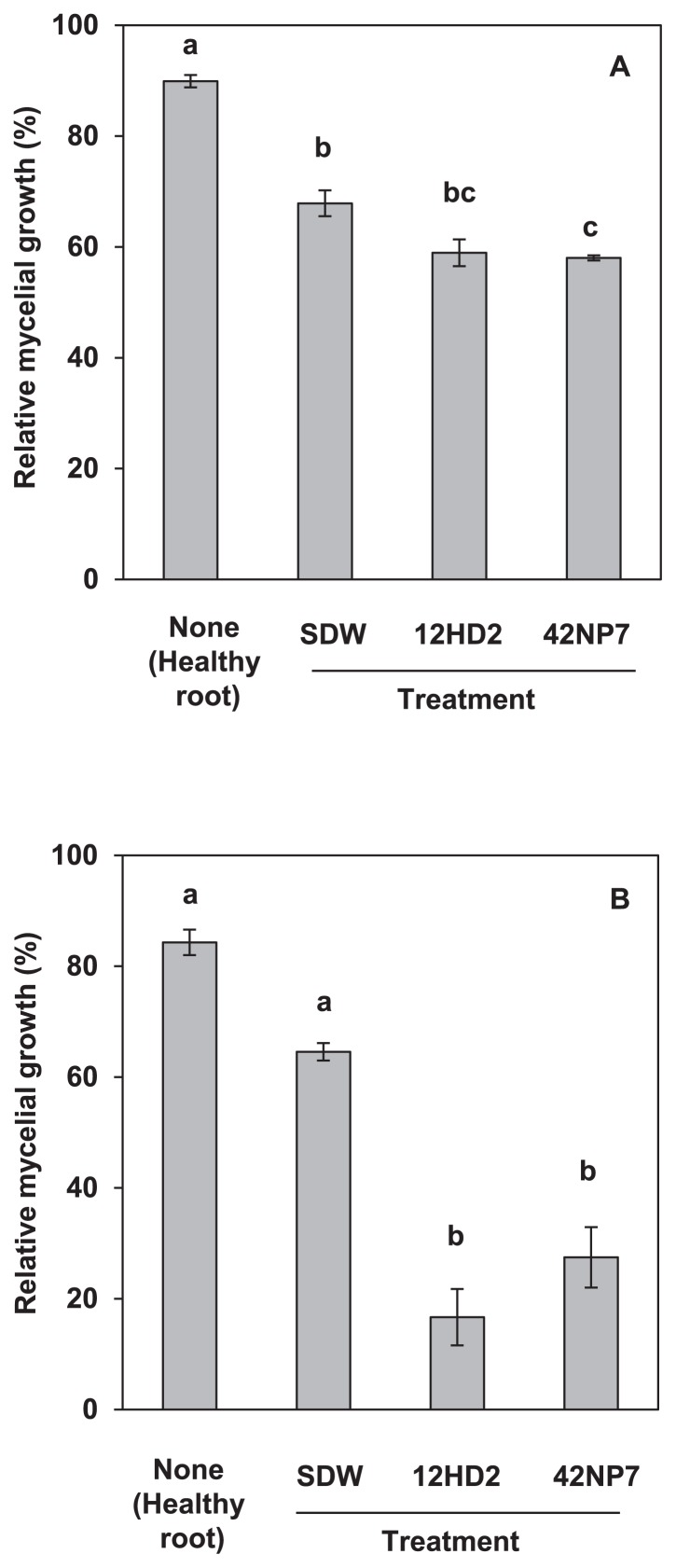
Antifungal activity in acetone extracts from the roots of tomato seedlings treated with bacterial cells of *Paenibacillus* strains 12HD2 and 42NP7 against FORL (A) and an indicator phytopathogenic fungus, *Colletotrichum dematium* (B). Antifungal activity was evaluated based on the relative diameter of mycelial growth from a small mycelial block of the fungus relative to growth following a treatment with a control solution (50% methanol). Each value indicates the mean of three experiments and bars denote the standard error of the mean. Different letters within each column indicate significant differences according to Fisher’s LSD test.

Fig. 7 shows the relative mycelial growth of FORL and *C. dematium* treated with acetone extracts from tomato roots with or without the bacterial treatment. DS values on the plant roots treated with *Paenibacillus* strains 12HD2 and 42NP7 were 48.6 and 37.8, respectively, while that of the SDW-treated roots was 68.3. Under such conditions, inhibition of the mycelial growth of FORL and *C. dematium*, based on the dropping assay, was slightly stronger in extracts obtained from roots treated with both *Paenibacillus* strains. Against FORL, the colony diameters of the extracts from untreated roots and SDW-treated roots were 89.9 and 67.9% that of the control (50% methanol), respectively, and the extract obtained from 42NP7-treated roots significantly (*P*<0.05) more reduced the diameter comparing to them (Fig. 7A), although the relative mycelial growth by that of 12HD2- treated roots was not significantly different. Similarly, the inhibitory effects of the extracts from both *Paenibacillus* strain-treated roots against *C. dematium* were 16.7 and 26.5% in the colony diameter of the relative control and the inhibition of growth was significantly stronger than that from untreated roots and SDW-treated roots (Fig. 7B).

### RT-PCR analysis

**Fig. 8:**
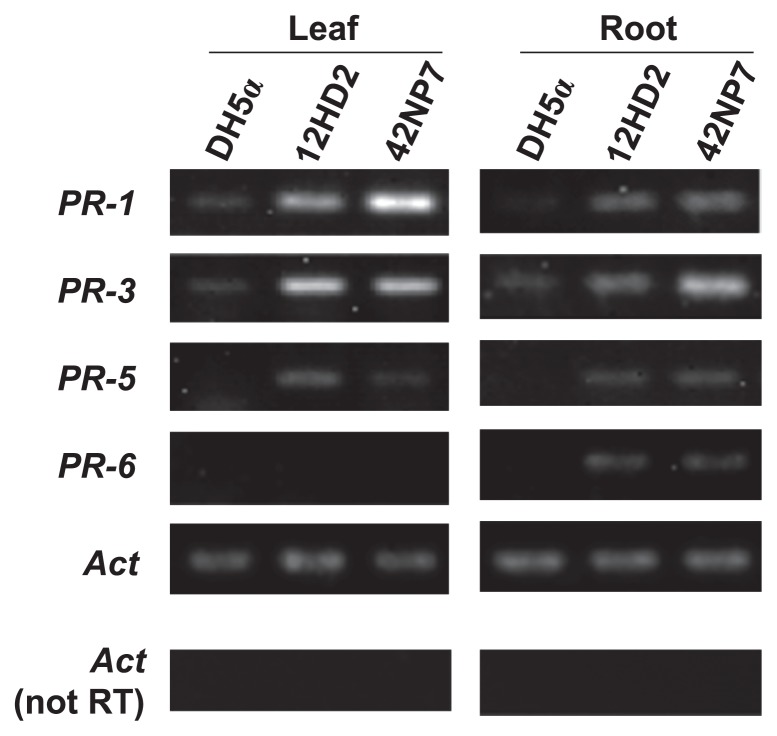
Expression of defense-related genes in the leaves and roots of tomato seedlings treated with the cells of *Paenibacillus* strains 12HD2 and 42NP7. SA-responsive (*PR-1*, *PR-5*) and JA/ET-responsive (*PR-3*, *PR-6*) genes were used as representative markers in RT-PCR. The expression of genes in the seedlings treated with *Escherichia coli* DH5α are displayed as a control. The actin gene, *Act*, was used as an internal standard for expression. *Act* (not RT) represents the amplification of *Act* using nonreverse-transcripted RNA as a negative control.

The expression of defense-related genes in tomato plants treated with each cell suspension of the *Paenibacillus* strains 12HD2 and 42NP7 is shown in Fig. 8. The expression levels of all the targeted genes were stronger on the roots following the treatment with both strains than on roots treated with DH5α as a control, and a clearer signal band on *PR-3* appeared following the treatment with 42NP7. The expression of *PR-1*, *PR-3*, and *PR-5*, but not *PR-6*, was also observed in leaf samples following the treatment with both strains.

## Discussion

Of 20 strains obtained from the tomato phyllosphere in the seedling experiment, two *Paenibacillus* strains, 12HD2 and 42NP7, were confirmed to significantly suppress FCRR. This result indicated that the phyllosphere harbors bacterial strains that have the potential to suppress soilborne FCRR. The suppressive activities of the selected strains were ascertained in potted and field experiments, and the population density of *F. oxysporum* (mostly FORL) was reduced to less than 80% of the control; therefore, these strains can be considered to have potential as biological control agents (BCAs) against the disease. In previous studies on biological control using *Paenibacillus* bacteria, *P. polymyxa* was shown to suppress Ralstonia wilt of tomato (1), crown rot of peanut (13), phytophthora blight of pepper plant (21), and the oomycete plant pathogens *Phytophthora palmivora* and *Pythium aphanidermatum* in an *Arabidopsis thaliana* model system (39). In addition, *P. alvae* was found to protect the eggplant, tomato, and potato against *Verticillium dahliae* (40). To the best of our knowledge, this is the first study to demonstrate the suppressive potential of bacterial strains belonging to *Paenibacillus* against FCRR. Additionally, these two candidate strains, 12HD2 and 42NP7, were taxonomically different from *P. polymyxa* and *P. alvae*, but were closely related to *P. alginolyticus* and *P. favisporus*, respectively, based on similarities in their 16S rRNA gene sequences. *P. alginolyticus* was originally obtained from soil (29, 36). Although *P. favisporus* has been obtained from the rhizosphere (14), this species was originally isolated from aged cow dung (43). These findings imply that both bacterial species have the potential to colonize both the phyllosphere and soil.

The suppressive activities of the selected *Paenibacillus* strains against the disease were found to occur following the application of the washed cell suspension, but not the culture filtrate (Fig. 3). In addition, neither strain displayed direct antifungal activity against FORL on both PDA and AR2A plates, which consisted of different ingredients (Fig. 4). These results suggested that both disease suppression and reductions in the FORL population in the root tissue following the treatment with these two strains were not based on the secretion of antifungal compounds. Although Saidi *et al.* (35) reported two *Bacillus* strains as BCA candidates against FCRR and showed antifungal activity against FORL, the causal factor of the suppressive activities of the *Paenibacillus* strains in this study appeared to differ between the *Bacillus* strains.

SEM observations revealed that the colonization of bacterial cells occurred on the surface of roots treated with cells of each *Paenibacillus* strain, with assemblages or clusters being formed particularly in the interspaces of epidermal root cells (Fig. 5). In addition, 42NP7 cells were detected in root tissue inoculated with the strain for 28 d (Fig. 6). These results suggested that suppressive activity may occur from the root colonization of these strains. The importance of the colonization of roots for the biological control of soilborne diseases has been well documented, particularly for several *Pseudomonas* strains (45). Regarding *Paenibacillus*, Haggag and Timmusk (13) reported that *P. polymyxa* colonized and formed biofilms on peanut roots, and suppressed crown root rot disease caused by *Aspergillus niger*. Similar associations between bacterial colonization and potential biocontrol activity were demonstrated in the present study.

Acetone extracts from both *Paenibacillus* strain-treated tomato roots exhibited stronger antifungal activities against FORL and the indicator fungus, *C. dematium* than those obtained from healthy roots (Fig. 7), although the activity of the extract obtained from 12HD2 treated-roots against FORL was not significantly different from that of the SDW-treated roots. Since antibiotic activity by the bacteria was absent *in vitro*, colonized bacterial cells may have induced the accumulation of antifungal compounds in the roots of the plant, leading to the suppression of the disease. We speculated that the main antifungal compound of the extracts may be *a*-tomatine, a tomato phytoanticipin, based on our preliminary experiments. Although antifungal activity was weaker against FORL than *C. dematium*, this may have been due to the secretion of tomatinase, an enzyme for the degradation of *a*-tomatine by FORL (19), while the production of enzymes by *C. dematium* has not yet been reported. Further investigations are needed to identify the antifungal compounds accumulated in the roots.

Together with the significant increase observed in the antifungal activity in the extracts obtained from *Paenibacillus-*treated roots, the colonization of these bacterial cells on roots may trigger the accumulation of antifungal compounds in plants as a defense response mechanism in the roots, leading to the suppression of FCRR. Furthermore, we analyzed the expression of defense-related genes in tomato plants by root treatments with each cell suspension of the two strains (Fig. 8), and found that the expression of SA-responsive genes (*PR-1* and *PR-5*) and JA/ET-responsive genes (*PR-3* and *PR-6*) were induced in the root tissue. Thus, both strains have the potential to accumulate defense-related proteins in the treated-root tissues, in addition to the accumulation of antifungal compounds, as above described, and may additively or synergistically play a suppressive role against the disease. The SA defense pathway, which plays a major role in the activation of defenses against biotrophic pathogens, and the JA/ET defense pathway, which is more commonly associated with defense against necrotrophic pathogen attacks, have generally been considered to work antagonistically (7, 26). Previous studies on the induction of plant defense pathways by *Paenibacillus* and *Bacillus* spp. (4, 18, 30, 34, 41) revealed that *P. alvae* strain K165 (41) and *B. subtilis* strain GB03 (34) activated the SA and ET pathways in *Arabidopsis thaliana*, respectively. On the other hand, Niu *et al.* (30) reported that *B. cereus* AR156 activated both of these pathways simultaneously. Similarly, the co-activation of both pathways following the treatment of the two selected *Paenibacillus* strains on the tomato roots was suggested in this study. Various compounds, such as volatile organic compounds produced by *Paenibacillus* and *Bacillus* spp., have been shown to elicit plant defense pathways (4, 10, 17, 24, 25, 34). In the present study, we determined no compound eliciting defense pathways in the tomato plants. We showed that the cellular fraction, but not the culture filtrate exhibited disease suppression activity, which indicated that a cellular component of the strains or a compound produced by the strains after the colonization on the roots activated the defense pathway. In addition to the enhanced expression of defense-related genes in the root, bacterial treatments induced the expression of SA-responsive genes (*PR-1* and *PR-5*) and the JA/ET-responsive gene (*PR-3*) in the second leaf tissues of tomato seedlings, which suggested that tomato plants developed systemic resistance due to the action of the *Paenibacillus* strains. Hence, these strains may suppress not only FCRR, but also several airborne diseases of the tomato, and experiments are ongoing to elucidate this possibility.

## Figures and Tables

**Table 1:** Bacterial strains (*Bacillus* and *Paenibacillus*) isolated from the tomato phyllosphere and used for seedling experiments

Strain^a^	Accession No.^b^	Closely related species^c^	Homology (%)^c^	Disease severity (DS)^d^	Protective value (PV)^e^
12HD2	AB242753	*Paenibacillus alginolyticus* DSM 5050	98.6	27.5 bc	40.6
12HD4	AB242667	*Bacillus niacini* IFO15566	98.8	33.4 abc	27.9
22HD1	AB242662	*Bacillus solisalsi* YC1	98.4	37.5 abc	18.9
22HD4	AB242763	*Paenibacillus xylanilyticus* XIL14	99.3	40.0 abc	13.6
31ND2	AB242757	*Paenibacillus kobensis* DSM 10249	95.8	34.5 abc	25.5
31NP3	AB242674	*Bacillus parviboronicapiens*^f^ BAM-582	97.7	39.2 abc	15.3
42ND16	AB242755	*Paenibacillus alginolyticus* DSM 5050	98.4	34.2 abc	26.1
42ND17	AB242663	*Bacillus weihenstephanensis* DSM11821	98.4	44.5 ab	3.9
42ND20	AB242668	*Bacillus foraminis* CV53	95.1	37.8 ab	18.3
42NP7	AB242671	*Paenibacillus favisporus* GMP01	99.7	22.5 c	51.4
42NP15	AB242664	*Bacillus megaterium* IAM 13418	99.6	36.7 abc	20.6
42NP17	AB242672	*Bacillus simplex* DSM 1321	99.8	42.2 abc	8.8
52HD3	AB242659	*Bacillus stratosphericus* 41KF2a	100	39.2 abc	15.3
62HD17	AB242675	*Bacillus mojavensis* IFO15718	99.7	35.9 abc	22.5
62ND1	AB181686	*Bacillus safensis* FO-036b	98.7	34.2 abc	26.1
62NP15	AB242758	*Paenibacillus lautus* JCM 9073	96.2	37.5 abc	19.0
62NP21	AB242756	*Paenibacillus glycanilyticus* DS-1	98.3	31.7 abc	31.6
83ND30	AB242669	*Bacillus panaciterrae* Gsoil 1517	99.5	36.7 abc	20.7
104NP2	AB242644	*Bacillus solisalsi* YC1	97.8	35.9 abc	22.5
104NP13	AB242645	*Bacillus benzoevorans* NCIMB 12555	97.4	30.0 abc	35.2
Control^g^				46.3 a	

a Each strain was obtained in a previous study (9).

b The accession number of the 16S rRNA gene sequence of each strain was deposited in the GenBank data library.

c Closely related species and its homology (%) in the library based on the gene sequence.

d Different letters indicate significant differences using Fisher’s LSD test (*P*<0.05).

e Calculation was performed based on the mean DS value.

f Present name is *Lysinibacillus parviboronicapiens*.

g Seedlings were treated with SDW as a control.

**Table 2:** Primers used for RT-PCR

Name	Sequence (5′→3′)	Target gene
LePRP6f	TGTCCGAGAGGCCAAGCTAT	*PR-1*
LePRP6r	AGGACGTTGTCCGATCCAGT	*PR-1*
LeChi9f	GACCATACGCATGGGGTTAC	*PR-3*
LeChi9r	CTCCAATGGCTCTTCCACAT	*PR-3*
LePRPA5f	AGGTCCATGTGGCCCTACTG	*PR-5*
LePRPA5r	TCACTTGAGGGCATCTCCAA(	*PR-5*
LeCEVI57Gf	TGTACGACGTGTTGCACTGG	*PR-6*
LeCEVI57Gr	TGCAACCCTCTCCTGCACTA	*PR-6*
LeActinF	GCCCCACCTGAGAGGAAGTA	*Act*
LeActinR	AGGGAGCTGCTCTGGAAATG	*Act*
